# Molecular and Structural Basis of the Proteasome α Subunit Assembly Mechanism Mediated by the Proteasome-Assembling Chaperone PAC3-PAC4 Heterodimer

**DOI:** 10.3390/ijms20092231

**Published:** 2019-05-07

**Authors:** Tadashi Satoh, Maho Yagi-Utsumi, Kenta Okamoto, Eiji Kurimoto, Keiji Tanaka, Koichi Kato

**Affiliations:** 1 Graduate School of Pharmaceutical Sciences, Nagoya City University, 3-1 Tanabe-dori, Mizuho-ku, Nagoya 467-8603, Japan; tadashisatoh@phar.nagoya-cu.ac.jp (T.S.); mahoyagi@ims.ac.jp (M.Y.-U.); kenta.okamoto@icm.uu.se (K.O.); 2 Exploratory Research Center on Life and Living Systems (ExCELLS), National Institutes of Natural Sciences, 5-1 Higashiyama, Myodaiji, Okazaki, Aichi 444-8787, Japan; 3 Institute for Molecular Science, National Institutes of Natural Sciences, 5-1 Higashiyama, Myodaiji, Okazaki, Aichi 444-8787, Japan; 4 Faculty of Pharmacy, Meijo University, Tempaku-ku, Nagoya 468-8503, Japan; kurimoto@meijo-u.ac.jp; 5 Laboratory of Protein Metabolism, Tokyo Metropolitan Institute of Medical Science, 2-1-6, Kamikitazawa, Setagaya-ku, Tokyo 156-8506, Japan; tanaka-kj@igakuken.or.jp

**Keywords:** assembly chaperone, molecular matchmaker, molecular modeling, proteasome, X-ray crystal structure

## Abstract

The 26S proteasome is critical for the selective degradation of proteins in eukaryotic cells. This enzyme complex is composed of approximately 70 subunits, including the structurally homologous proteins α1–α7, which combine to form heptameric rings. The correct arrangement of these α subunits is essential for the function of the proteasome, but their assembly does not occur autonomously. Assembly of the α subunit is assisted by several chaperones, including the PAC3-PAC4 heterodimer. In this study we showed that the PAC3-PAC4 heterodimer functions as a molecular matchmaker, stabilizing the α4-α5-α6 subcomplex during the assembly of the α-ring. We solved a 0.96-Å atomic resolution crystal structure for a PAC3 homodimer which, in conjunction with nuclear magnetic resonance (NMR) data, highlighted the mobility of the loop comprised of residues 51 to 61. Based on these structural and dynamic data, we created a three-dimensional model of the PAC3-4/α4/α5/α6 quintet complex, and used this model to investigate the molecular and structural basis of the mechanism of proteasome α subunit assembly, as mediated by the PAC3-PAC4 heterodimeric chaperone. Our results provide a potential basis for the development of selective inhibitors against proteasome biogenesis.

## 1. Introduction

The selective degradation of proteins in eukaryotic cells is essential for the maintenance of physiological homeostasis. Protein degradation is implemented primarily via the ubiquitin-proteasome system [1,2]. The proteasome is huge protein complex (26S), comprised of a 20S core particle (CP) and one or two 19S regulatory particles (RPs). The 20S CP, which has proteolytic activity, is composed of seven homologous *α* subunits, α1–α7, and seven homologous β subunits, β1–β7, which are assembled into a cylindrical structure with an α_1-7_β_1–7_β_1–7_α_1–7_ arrangement. The 19S RP is responsible for the collection of ubiquitinated substrates, the opening of the central gating pore of the 20S CP, and the de-ubiquitination and translocation-coupled unfolding of the substrates. Recent structural studies using cryo-electron microscopy have shed light upon the cooperative working mechanisms of this huge proteolytic machinery [3,4].

The correct arrangement of the proteasomal subunits is essential to the proper functioning of eukaryotic proteasomes. There is considerable evidence that the assembly of the eukaryotic 26S proteasome does not proceed spontaneously, but is mediated by several assembly chaperones [5,6,7,8]. The formation of the 20S CP is assisted by five proteasome-specific chaperones: PAC1–PAC4 and POMP in humans; Pba1–Pba4 and Ump1 in yeast. Four dedicated chaperones, p27 (Nas2), gankyrin (Nas6), PAAF1 (Rpn14), and S5b (Hsm3) are responsible for the formation of the base subcomplex of the 19S RP. Malfunctions of these assembly chaperones cause the accumulation of imperfectly assembled or mis-assembled complexes of the proteasomal subunits. For example, knock-down experiments involving PAC3 and PAC4 results in the accumulation of abnormal α-subunit oligomers lacking the α3–α7 subunits [9,10].

Because proteasome biogenesis is known to be significantly upregulated in cancer cells [11], the proteasome has potential as a target for therapeutic drugs for cancer treatment [12,13]. Bortezomib (Velcade) has been widely used as proteasome inhibitor for the treatment of patients with multiple myeloma [14]. The chaperones involved in proteasome assembly have also been considered as potential drug targets for anticancer treatments [12]. Selective inhibitors that specifically suppress proteasome biogenesis could be valuable for minimizing the undesired side effects which can occur when using compounds which target mature proteasomes.

Recently reported knock-out experiments indicated that α4, α5, α6, and α7 form a core assembly intermediate as part of the initial process of α-ring assembly, which is supported by PAC3-PAC4 [15]. However, most of the biochemical and structural data about the proteasome-assembly chaperones have been generated mainly from yeast proteins, which have only modest sequence identities with the human counterparts; less than 20%, for PAC3 when compared with Pba3. As with CP-assembly, yeast Pba3 and Pba4 have structural resemblance, and form a heterodimer [16] which functions as a matchmaker mediating the association between α4 and α5 [17]. It remains unclear, however, how the human PAC3-PAC4 complex functions in α-ring assembly through specific, direct interactions with cognate proteasomal subunits, although the crystal structures of human PAC3 and PAC4 have been solved for their homodimeric forms [16,18].

Structural insights into the chaperone-mediated formation of the human proteasome are important for the design and development of low-toxicity anticancer drugs which can inhibit the protein-protein interactions involved in the proteasome-assembly process. We performed a biochemical and biophysical study of the human PAC3-PAC4 heterodimer in order to understand the functional and structural mechanisms of α-ring formation mediated by the proteasome-assembling chaperones.

## 2. Results and Discussion

### 2.1. The PAC3-PAC4 Heterodimer Interacts Primarily with α5

To study the biochemical processes involved in proteasome α-subunit assembly mediated by the PAC3-PAC4 heterodimer, we prepared all of the human proteasome α subunits as recombinant proteins. Although protocols to prepare PAC3 and PAC4 as individual recombinant proteins have been reported previously, their heterodimer is rather unstable, unlike the yeast orthologs Pba3 and Pba4 [16,18]. The recombinant PAC4 also has a tendency to form a domain-swapped homodimer [18]. To overcome these problems, we designed and prepared a PAC3-PAC4 heterodimer as a single-chain form, termed scPAC3/4, in which the C-terminus of PAC4 is connected to the N-terminus of PAC3 via a (GGGS)_4_ liner. All of the recombinant proteins were produced using bacterial expression systems in *Escherichia coli*, and were successfully purified to homogeneity (Figure S1).

**Figure 1:**
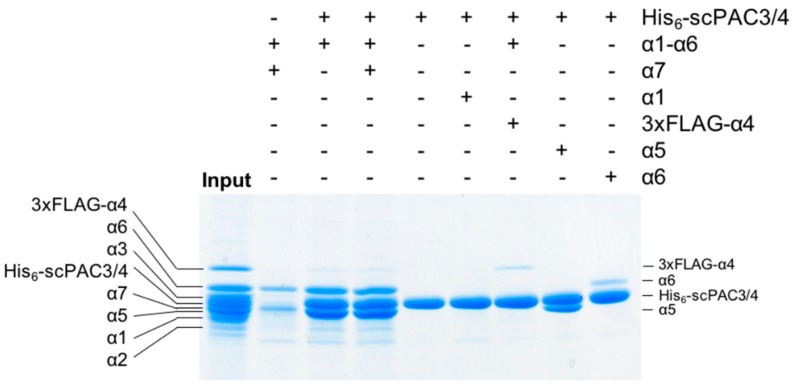
Pull-down experiments between the PAC3-PAC4 heterodimer and proteasome α subunits. The non-tagged α1–α3 and α5–α7 along with 3xFLAG-tagged α4 were mixed with His_6_-tagged scPAC3/4 immobilized on Ni^2+^-charged Chelating Sepharose beads. The 3xFLAG-tagged α4 was used to avoid the band overlap between α4 and scPAC3/4. After extensive washing, bound proteins were analyzed using CBB staining after sodium dodecyl sulfate polyacrylamide gel electrophoresis (SDS-PAGE). The ‘Input’ lane contained all α subunits and His_6_-scPAC3/4 (0.5 μg each). The SDS-PAGE bands were assigned according to Figure S1a, and the bands originating from the His_6_-scPAC3/4 and the bound α subunits are labeled.

To determine which proteasomal *α* subunits interact with the PAC3-PAC4 heterodimer, we performed in vitro pull-down experiments using these recombinant proteins. In the pull-down assay, His_6_-tagged scPAC3/4 was applied to Ni^2+^-charged resin, and subsequently incubated with a mixture of all of the α-subunit proteins. Since α7 spontaneously forms an oligomer that is capable of capturing α6 [19,20], we carried out this experiment both in the absence and in the presence of α7. The pull-down experiments showed that scPAC3/4 reacted with several α subunits including α4, α5, and α6 (Figure 1). Addition of α7 had virtually no impact on the interaction of α6 with scPAC3/4, suggesting that it has a higher affinity for the PAC3-PAC4 heterodimer than for the α7 oligomer. To avoid ambiguity due to overlapping of the Coomassie Brilliant Blue (CBB)-stained bands, we performed the pull-down experiments using α1, α4, α5, and α6 individually. The pull-down assay showed that scPAC3/4 interacted most strongly with α5 and weakly with α4 and α6. By contrast, no interaction was detected between scPAC3/4 and α1. The interaction between α6 and scPAC3/4 appeared to be enhanced in the presence of the other α subunits. Since α5 and α6 occur consecutively in the native α ring, these data suggest that the PAC3-PAC4 heterodimer is important for α5-α6 subcomplex assembly during proteasome α-ring formation.

### 2.2. The PAC3-PAC4 Heterodimer Acts as Molecular Matchmaker in α4-α5-α6 Assembly

**Figure 2:**
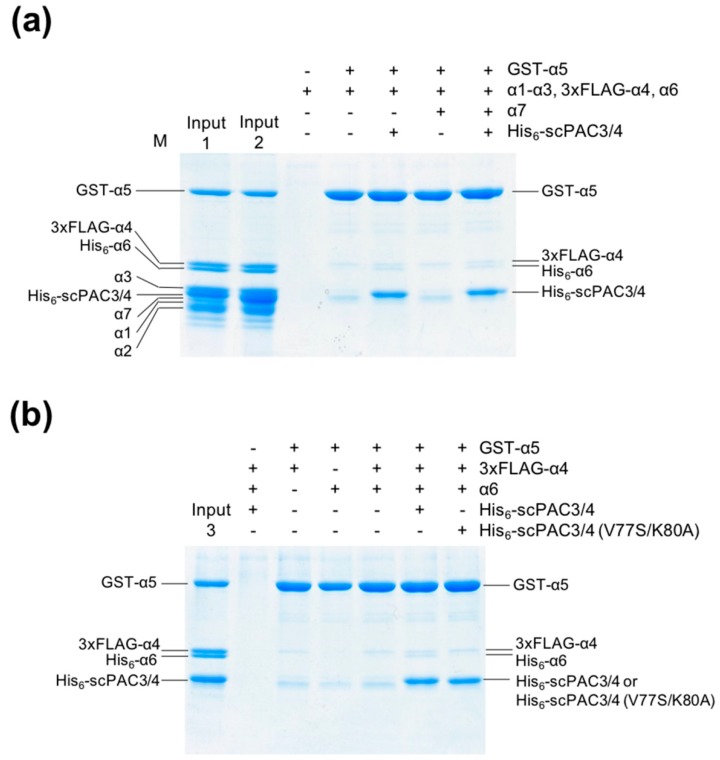
Pull-down experiments between α5 and the other α subunits. The α1–α4, α6, and α7 subunits were mixed with GST-tagged α5 immobilized on Glutathione Sepharose beads. The 3xFLAG-tagged α4 and His_6_-tagged α6 were used to avoid the overlap of their bands with those of the other α subunits or scPAC3/4. (**a**) Interactions between α5 and the other α subunits in the presence and absence of the PAC3-PAC4 heterodimer. The ‘Input1′ and ‘Input2′ lanes contained His_6_-scPAC3/4 and α1–α6 subunits in the absence and presence of α7, respectively (0.5 μg each). (**b**) Interaction between α5 and the adjacent α subunits, α4 and α6. The ‘Input3′ lane contained His_6_-scPAC3/4 and α4–α6 subunits. The pull-down experiment was also performed using an scPAC3/4 mutant with V77S and K80A substitutions in PAC3. Band assignments were carried out according to Figure S1b.

In order to explore the functional mechanism of the PAC3-PAC4 heterodimer in proteasome assembly involving α4–α6, we investigated the inter-subunit interactions mediated by scPAC3/4. In a pull-down assay, glutathione *S*-transferase (GST)-fused α5 was used as a bait for probing its interactions with the other α subunits, both in the absence and in the presence of scPAC3/4. GST-α5 interacted weakly with α6 in the presence of scPAC3/4, while the other subunits were not reactive with α5 regardless of the presence or absence of scPAC3/4 (Figure 2). In contrast, GST-α5 weakly interacted with α4 regardless of the presence or absence of scPAC3/4 under this assay condition. The results were not influenced by the presence of α7 (Figure 1 and Figure 2).

In yeast, the Pba3-Pba4 heterodimer acts as a matchmaker, reinforcing interactions between the α4 and α5 subunits [17]. The results of our pull-down analysis indicated that the PAC3-PAC4 heterodimer interacts with α4, α5, and α6, thereby acting as a molecular matchmaker for these proteasomal subunits. These findings suggest that the functional roles and interactions of this assembly chaperone complex with the proteasomal subunits are evolutionally conserved between yeast and humans.

### 2.3. Structural Insights into the Mechanisms Underlying PAC3/PAC4-Dependent α4-α5-α6 Assembly

To investigate the structural mechanisms underlying the chaperone-dependent formation of the α4-α5-α6 subcomplex, we built a three-dimensional model of the putative quintet complex comprised of PAC3, PAC4, α4, α5 and α6, using previously-reported crystallographic data. Crystal structures for the PAC3 homodimer [16], domain-swapped PAC4 homodimer [18], and 20S proteasome [21] have been published. In addition, we newly determined a 0.96-Å high-resolution trigonal structure of the PAC3 homodimer (Figure S2a). The overall structure of the trigonal form was very similar to that of the tetragonal structure we have previously published, except for a loop comprised of residues 51–61 (Figure S2b), suggesting that it is mobile. Loop flexibility was also observed in the corresponding segment of the yeast ortholog Pba3 in its heterodimer with Pba4 [16] (Figure S3). Our nuclear magnetic resonance (NMR) relaxation data from the human PAC3 homodimer confirmed that the loop is indeed mobile and disordered in solution (Figure S4).

**Figure 3:**
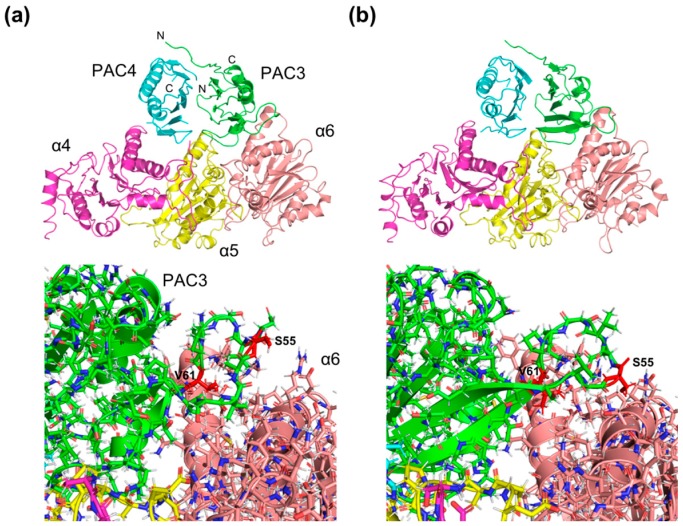
Three-dimensional model of the quintet complex comprising PAC3, PAC4, α4, α5, and α6. (**a**) Complex model A, based on the 2.00-Å PAC3 structure. (**b**) Complex model B, based on the newly-determined 0.96-Å structure. The positions of the N- and C-termini are indicted. Overall and close-up views between PAC3 and α6 of the quintet-complex models are shown in the upper and lower parts of the figure, respectively. Putative α6-binding residues of PAC3, Ser55 and Val61 (see also Figure 4b), are highlighted in red in both models to highlight the conformational differences of the loop between the two models.

In the quintet-complex models, in addition to the interactions between PAC3-PAC4 and α5, based on the crystal structure of the yeast counterparts [16], the assembly chaperone interacted with the neighboring α4 and α6 subunits (Figure 3a). When the PAC3-4/α4/α5/α6 quintet complex model was superimposed onto the crystal structure of 20S CP, PAC3 and PAC4 make steric hindrance with β6 and β5, respectively, which possibly triggers the release of PAC3-4 from the α-ring upon binding of the β subunits onto the α-ring. A complex model, model A, based on the 2.00-Å PAC3 structure showed that the mobile loop was turned toward the solvent. Another model based on the 0.96-Å structure, model B, showed that the corresponding loop contacts α6. Apart from interactions involving this mobile loop, intermolecular contacts between the PAC3-PAC4 heterodimer and the proteasomal subunits are almost identical in the two models. Therefore, in the rest of this paper, we discuss the structural basis of the PAC3/PAC4-dependent α4-α5-α6 subunit assembly using model B.

**Figure 4:**
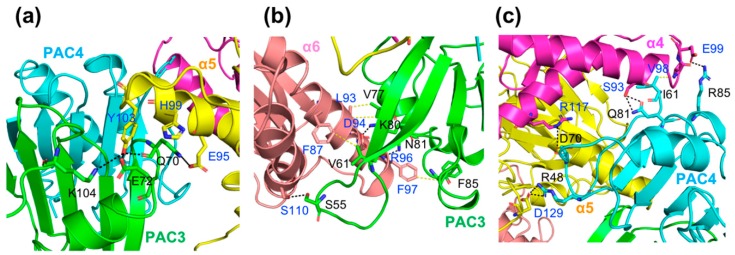
Predicted interaction interfaces between the PAC3-PAC4 heterodimer and proteasomal α4-α5-α6 subunits. (**a**) PAC3-α5. (**b**) PAC3-α6. (**c**) PAC4-α4 or α5 interfaces. Residues involved in the interactions are shown as stick representations. Potential hydrogen bonds and non-polar interactions are indicated as black and yellow dotted lines, respectively.

In this model, the interaction of α5 with the PAC3-PAC4 heterodimer is mediated by Gln70, Glu72, and Lys104 in PAC3, Arg48 in PAC4, and Glu95, His99, Tyr103, and Asp129 in α5 through electrostatic interactions and hydrogen bonds (Figure 4). Our pull-down data indicated that scPAC3/4 interacted most strongly with α5 and weakly with α4 and α6 (Figure 1). The model predicted additional interactions between PAC3 and α6, and between PAC4 and α4 (Figure 4b,c). Specifically, Ser55, Lys80, and Asn81 of PAC3 form hydrogen bonds or electrostatic interactions with Ser110, Asp94, and Arg96 of α6, respectively. There are also predicted hydrophobic interactions of Val61, Phe85, and Val77 in PAC3 with Phe87, Phe97, and Leu93 in α6. Additionally, Asp70 and Arg85 of PAC4 have electrostatic interactions with Arg117 and Glu99 of α4, respectively (Figure 4c). Gln81 and Ile61 of PAC4 form hydrogen bonds and hydrophobic interactions with Ser93 and Val98 of α4, respectively.

To validate our docking model, we performed mutational experiments, especially focusing on the interaction between PAC3 and α6, which were specifically observed in the human proteins as compared with yeast counterparts. We constructed an scPAC3/PAC4 mutant in which putative α6-binding residues, Val77 and Lys80, of PAC3 are replaced with Ser and Ala, respectively. As expected, our mutational analysis indicated that mutations of Val77 and Lys80 of PAC3 impaired interaction with α6 but not with α4 and α5 (Figure 2b), confirming the validity of our docking model.

Although the overall structures of human PAC3 and PAC4 are similar to those of yeast Pba3 and Pba4 (RMSD = 1.9–2.1Å and 1.9–2.2 Å) respectively, their amino acid sequence similarities are low (PAC3 versus Pba3 11.0%; PAC4 versus Pba4 14.6%). The *α*-subunit contacting residues of human PAC3 and PAC4, as predicted by the model, are not well-conserved in the yeast orthologs Pba3 and Pba4 (Figure S5). Nevertheless, our model predicts that the complementarity at the interaction interfaces between the PAC3-PAC4 heterodimer and the proteasomal α4-α5-α6 subunits can be conserved in the yeast counterparts with a few exceptions. Perhaps the best example is the replacement of electrostatic interactions between Glu72 of PAC3 and His99 of *α*5 by non-polar contacts between Ala105 of Pba3 and Gln114 of *α*5. Therefore, despite the low sequence similarity, the overall interaction modes of the matchmaking chaperones with the proteasomal subunits appear to be conserved between humans and yeast. It is plausible that the conformational flexibility of the mobile 51–61 loop of PAC3, which carries the α6-contacting residues, contributes to the interaction adjustability.

In summary, we produced structural insights into the functional mechanisms of the PAC3-PAC4 heterodimer as a molecular matchmaker underpinning the α4-α5-α6 subcomplex during α-ring formation. These findings offer potential new approaches to the design of inhibitors against the protein-protein interactions involved in proteasome biogenesis.

## 3. Methods

### 3.1. Sample Preparation

Human proteasome α6 short isoform and α7 subunits were produced and purified as previously described [22,23]. Genes encoding the proteasome α1 and α4 subunits were subcloned into *Nde*I and *Sal*I sites in pET28b, and the α2 gene was inserted into the pRSFDuet-1 vector using *Nde*I and *Xho*I restriction enzyme sites (Merck Millipore, Burlington, MA, USA). As for α4, 3xFLAG sequence (DYKDHDGDYKDHDIDYKDDDDK) was added at the N-terminus. The α3 and α5 genes were subcloned into the *Bam*HI and *Xho*I or *Sal*I sites of modified pCold-I and pCold-GST vectors (TaKaRa Bio Inc., Kusatsu, Japan), respectively, in which a factor Xa cleavage site was replaced with that of TEV protease. The PAC3 and PAC4 genes were subcloned into *Nde*I and *Xho*I sites in pET28b, in which the C-terminus of PAC4 was connected to the N-terminus of PAC3 through a (GGGS)_4_ liner. Standard polymerase chain reaction method was used to generate a V77S/K80A PAC3 mutant. *Escherichia coli* BL21-CodonPlus (DE3)-RIL (Agilent Technologies, Santa Clara, CA, USA) was used for all recombinant protein expression.

For the expression of recombinant proteins, the *E. coli* cells were grown in LB medium containing kanamycin or ampicillin. Briefly, the recombinant proteins were purified from the soluble fractions, except for α2, which was purified from the inclusion bodies and refolded using standard dilution methods.

Purification of these recombinant proteins was performed using affinity chromatography with Anti-FLAG M2 Affinity gel (Sigma-Aldrich, St. Louis, MO, USA), Ni^+^-charged Chelating Sepharose, or Glutathione Sepharose 4B, anion-exchange chromatography with RESOURCE Q resin, and size exclusion chromatography with Superdex 75 pg or 200 pg resins (GE Healthcare, Chicago, IL, USA). For NMR analyses, the PAC3 homodimer was expressed in *E. coli* cells which were grown in M9 minimal medium containing [^13^C]glucose (2.0 g/L) and/or [^15^N]NH_4_Cl (1.0 g/L), and purified using a previously-described protocol [12].

### 3.2. Pull-Down Experiments

The 3xFLAG-tagged α4, GST-fused proteasome α5-subunit (GST-α5), non-tagged or His_6_-tagged forms of proteasome α1, α2, α3, α6, and α7 subunits, and scPAC3/4 were used in the pull-down assays. For immobilization, 20 μg of His_6_-tagged scPAC3/4 or GST-α5 was applied to Ni^2+^-charged Chelating Sepharose or Glutathione Sepharose 4B (GE Healthcare) resins, respectively. The His_6_-scPAC3/4-immobilized resins were incubated with 50 μg of α1–α7 subunits for 2 h at 4 °C in an incubation buffer (20 mM Tris-HCl (pH 8.0) and 150 mM NaCl). For α5, the GST-α5-immobilized resins were incubated with 50 μg of α1–α4, α6, and α7 in the presence and absence of 50 μg of scPAC3/4 as described above. Since α7 makes a stable complex with α6 [19,20], the pull-down experiments containing α7 were performed separately. The resins were washed four times with the incubation buffer, which contains 60 mM imidazole in the His_6_-tag pull-down assays. Proteins bound to the Chelating or Glutathione Sepharose resins were eluted using 20 mM Tris-HCl (pH 8.0)/500 mM imidazole or 50 mM Tris-HCl (pH 8.0)/10 mM reduced glutathione, respectively, and analyzed by SDS-PAGE, stained with CBB.

### 3.3. Crystallization, X-ray Data Collection, and Structure Determination

For crystallization, purified non-tagged PAC3 homodimer was produced at a concentration of 8.0 mg/mL in 20 mM Tris-HCl (pH 7.5) and 150 mM NaCl. Crystals were obtained in a buffer containing 30% PEG2000 monomethyl ether and 0.1 M potassium thiocyanate with incubation at 20 °C for three to four days. Crystals were transferred into the reservoir solution and flash-cooled in liquid nitrogen. Diffraction intensities were integrated using XDS [24] and data scaling was carried out using AIMLESS [25]. The crystals of PAC3 belonged to space group *P*3_1_21 and diffracted up to a resolution of 0.96 Å.

The trigonal structure of PAC3 was solved by the molecular replacement method using MOLREP [26], using the previously-reported tetragonal structure (PDB code 2Z5E) [16] as a search model. Automated model building and manual model fitting to electron density maps were performed using ARP/wARP [27] and COOT [28], respectively. Model refinement was carried out using REFMAC5 [29], and structure validation was conducted using MolProbity [30]. The data collection and refinement statistics of the PAC3 homodimer are summarized in Table S1. The molecular graphics were prepared using PyMOL (Schrödinger, New York, NY, USA).

### 3.4. Computer-Aided Model Building

The quintet-complex model comprising PAC3, PAC4, α4, α5, and α6 was created by several rounds of superimpositions using the coordinates of the human PAC3 homodimer (PDB codes, 2Z5E [16], and 6JPT (from this study)), the human PAC4 homodimer (PDB code: 5WTQ) [18], the human 20S proteasome (5LE5) [21], the yeast Pba3-Pba4 heterodimer (2Z5B) [16], and the yeast Pba3-Pba4/α5 ternary complex (2Z5C) [16]. The human PAC3-PAC4 heterodimer was created by superimposition of PAC3 and PAC4 monomers onto yeast Pba3 and Pba4, respectively. The resulting PAC3-PAC4 model was superimposed onto the yeast Pba3-Pba4 structure complexed with α5. Finally, to make a quintet complex model, the PAC3-PAC4-α5 (yeast) model was superimposed onto the human *α*5 subunit of the 20S proteasome. Subsequent protonation and energy minimization was performed using the CHARMm force field with the Discovery Studio program suite [31] (BIOVIA, San Diego, CA, USA).

### 3.5. NMR Spectroscopy

^13^C- and ^15^N-labeled non-tagged PAC3 homodimer (0.3 mM) and ^15^N-labeled non-tagged PAC3 homodimer (0.1 mM), dissolved in PBS (pH 6.8) containing 10% D_2_O (*v*/*v*), 1 mM EDTA, and 0.01% NaN_3_, were used for spectral assignment and relaxation experiments. All NMR data were acquired at 303 K using DMX-500, AVANCE-500, and AVANCE-800 spectrometers equipped with a 5-mm triple-resonance cryogenic probe (Bruker, Billerica, MA, USA). The NMR data were processed using TOPSPIN (Bruker) and NMRPipe [32]. Conventional 3D NMR experiments [33] were carried out for chemical shift assignments of the heteronuclear single-quantum correlation (HSQC) peaks originating from the PAC3 homodimer. Spectral assignments were carried out using SPARKY [34] and CCPNMR [35] software. ^15^N relaxation parameters, *T*_1_, *T*_2_, and ^15^N-^1^H heteronuclear nuclear Overhauser effect (NOE) were obtained at 303 K using an AVANCE-800 spectrometer and analyzed using the Protein Dynamics software in the Dynamics Center (Bruker).

### 3.6. Accession Numbers

The coordinates and structural factors of the crystal structure of the PAC3 homodimer have been deposited in the Protein Data Bank under accession number 6JPT. Backbone ^1^H and ^15^N chemical shift data of the PAC3 homodimer have been deposited in the Biological Magnetic Resonance Data Bank under accession number 27844.

## Supplementary Materials

Supplementary materials can be found at https://www.mdpi.com/1422-0067/20/9/2231/s1.

## Abbreviations

CBB	Coomassie Brilliant Blue
CP	Core particle
GST	Glutathione *S*-transferase
HSQC	Heteronuclear single-quantum correlation
NMR	Nuclear magnetic resonance
NOE	Nuclear Overhauser effect
PAC	Proteasome-assembling chaperone
RP	Regulatory particle
SC	Single-chain
SDS-PAGE	Sodium dodecyl sulfate polyacrylamide gel electrophoresis
