# Supplementary material for: Molecular and Structural Basis of the Proteasome α Subunit Assembly Mechanism Mediated by the Proteasome-Assembling Chaperone PAC3-PAC4 Heterodimer
Source: Int J Mol Sci. 2019 May 7;20(9):2231. doi: 10.3390/ijms20092231 (PMC6539346; doi:10.3390/ijms20092231)
Supplement: Supplementary file 1 [file ijms-20-02231-s001.pdf]

Supplementary Material

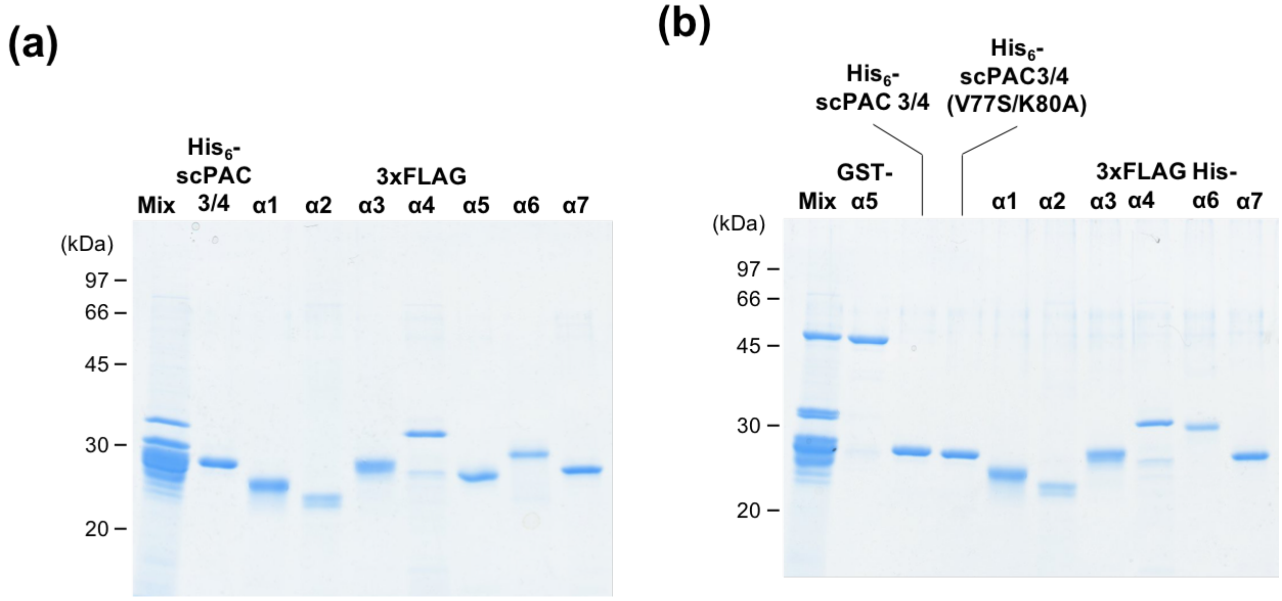

Figure S1. SDS-PAGE of purified recombinant human proteasome α1–α7 subunits and the assembly chaperone PAC3-PAC4 heterodimer

(a) His_6-tagged single-chain PAC3-PAC4 (scPAC3/4), 3xFLAG-tagged α4, and non-tagged α1–α3 and α5–α7 (0.5 μg each) used for the His_6-tag pull-down assay (Fig. 1). (b) GST-tagged α5, His_6-tagged wild-type and V77S/K80A scPAC3/4, 3xFLAG-tagged α4, His_6-tagged α6, and non-tagged α1–α3 and α7 (0.5 μg each) used for the GST pull-down assay (Fig. 2). The 'Mix' lane contained all α subunits and His_6-scPAC3/4.

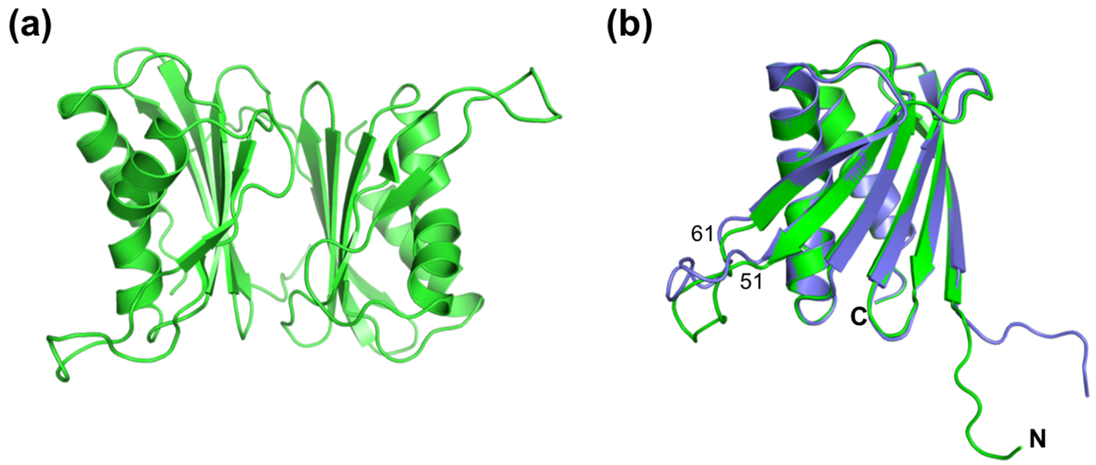

Figure S2. Crystal structure of PAC3 homodimer

(a) Overall structure of the 0.96-Å crystal structure of the PAC3 homodimer. One PAC3 molecule is contained in the crystallographic asymmetric unit and the symmetrically neighboring molecule is represented; (b) Comparison between the 0.96-Å trigonal (green) and 2.00-Å tetragonal (slate, PDB code: 2Z5E) crystal structures of the PAC3 homodimer.

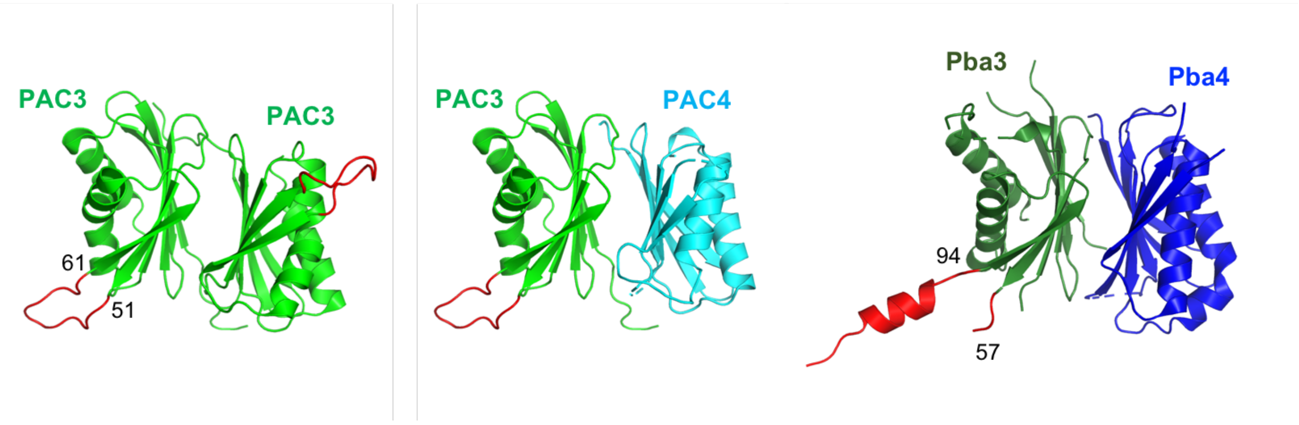

Figure S3. Comparison of crystal structures of the PAC3 homodimer and the Pba3-Pba4 heterodimer and the docking model of PAC3-PAC4 heterodimer

Superimposed PAC3 homodimer, PAC3-PAC4 heterodimer, and Pba3-Pba4 heterodimer (2Z5B) are shown in left, center, right, respectively. The flexible loop of PAC3 comprised of residues 51–61 and the corresponding loop of Pba3 are highlight in red.

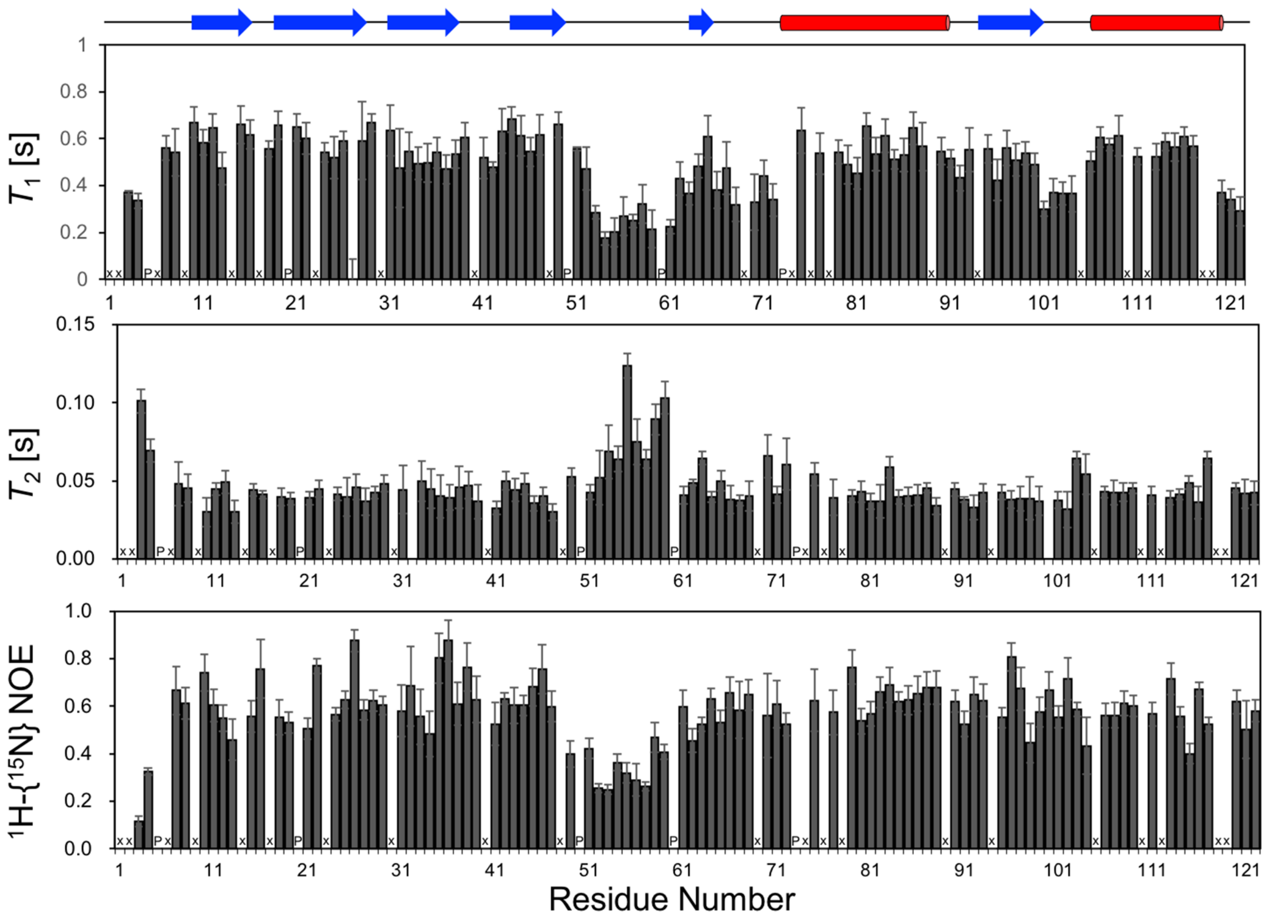

Figure S4. Backbone dynamics of the PAC3

Backbone ^{15}N T_1 longitudinal relaxation times (top), T_2 transverse relaxation times (middle), and heteronuclear ^{1}H-{^{15}N} NOE enhancements were plotted for each residue of the protein. Error bars for the T_1 and T_2 data denote curve-fitting uncertainties. Errors in the ^{1}H-{^{15}N} NOEs were estimated from the signal-to-noise ratio of the spectra. "P" and "x" indicate proline and unassigned residues, respectively. Secondary structure elements of PAC3 are indicated by red cylinder (α-helix) and blue arrow (β-strand) above the amino acid sequence.

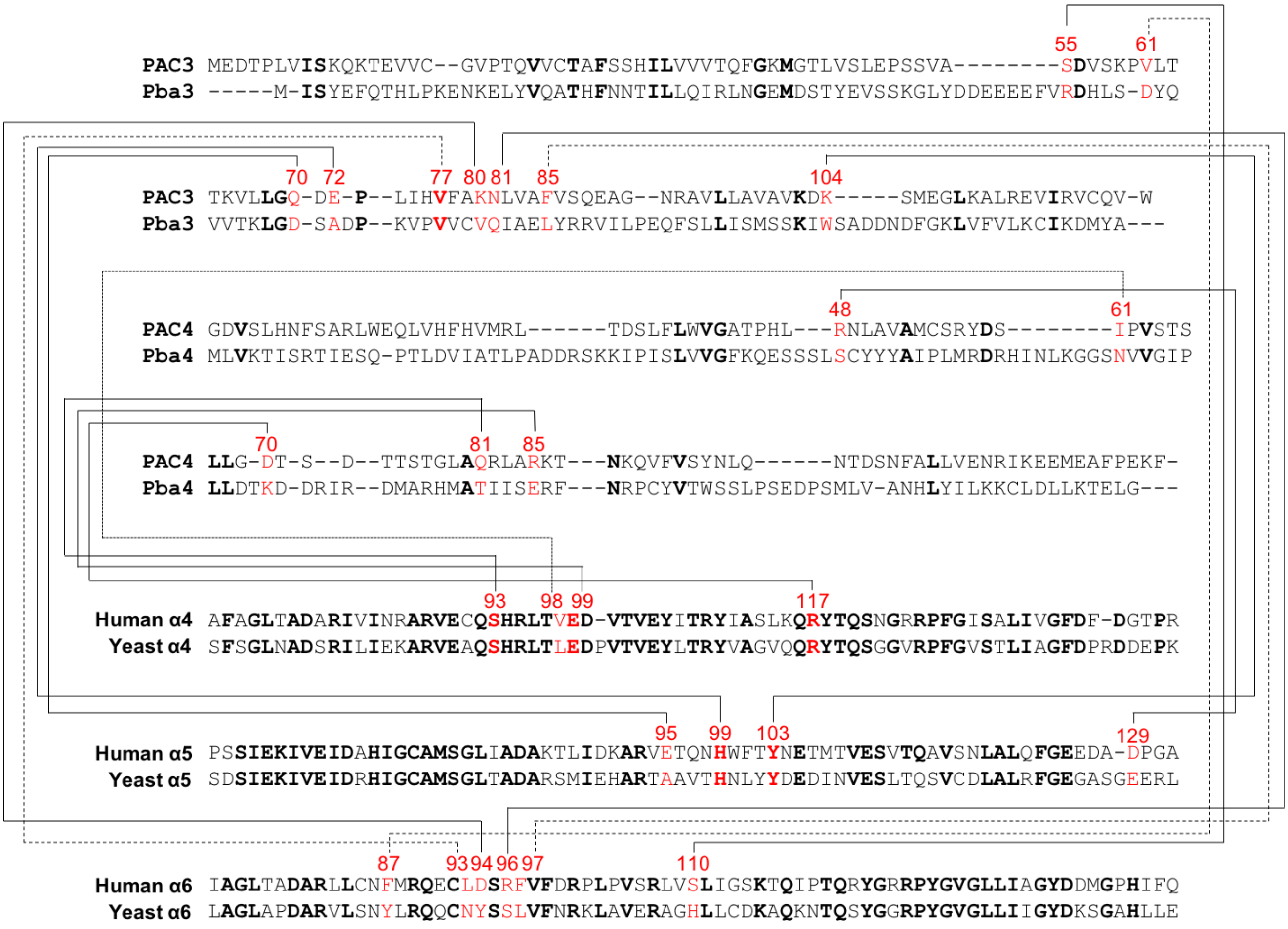

Figure S5. Structure-based sequence alignment of PAC3, Pba3, PAC4, and Pba4

The amino acid sequences were aligned using the program MATRAS[1]. Putative interacting residues in the PAC3/PAC4/α4/α5/α6 quintet-complex model are highlighted in red letters, along with the corresponding residues of the yeast counterparts. Identical residues of the assembly chaperones and proteasome α4–α6 subunits between humans and yeast are highlighted in bold letters. Solid and broken lines indicate predicted polar and non-polar interactions, respectively.

1. Kawabata, T. MATRAS: A program for protein 3D structure comparison. *Nucleic Acids Res.* 2003, *31*, 3367–3369)

Table S1. Data collection and refinement statistics for human PAC3 homodimer

	Human PAC3 homodimer
**Crystallographic data**	
Space group	$P3_12_1$
Unit cell *a*/*b*/*c* (Å)	71.2/71.2/47.1
**Data processing statistics**	
Beam line	SPring-8 BL44XU
Wavelength (Å)	0.90000
Resolution (Å)	47.07–0.96 (0.98–0.96)
Total/unique reflections	537,273/83,927
Completeness (%)	99.9 (98.0)
R_{merge} (%)	5.2 (116.2)
R_{meas} (%)	5.6 (130.3)
R_{pim} (%)	2.1 (57.9)
Multiplicity	6.4 (4.8)
Mean $CC_{1/2}$	0.998 (0.606)
Mean $I/\sigma(I)$	17.1 (1.3)
**Refinement statistics**	
Resolution (Å)	14.58–0.96
R_{work}/R_{free} (%)	13.6/14.6
RMS deviations from ideal	
Bond lengths (Å)	0.008
Bond angles (°)	1.05
Ramachandran plot (%)	
Favored	97.48
Allowed	2.52
Outliers	0.0
